# Cancer Immunomodulatory Effect of *Bidens pilosa* L. in Mice: Suppression of Tumor-Associated Macrophages and Regulatory T Cells

**DOI:** 10.3390/cells15020126

**Published:** 2026-01-10

**Authors:** Meihua Zhu, Jiayan Xiong, Ruyi Zhang, Xingyan Yang, Weiqing Sun, Ziyi Yang, Yuhan Chai, Yang Tao, Yu-Qiang Zhao, Baomin Fan, Guangzhi Zeng

**Affiliations:** 1 Yunnan Key Laboratory of Chiral Functional Substance Research and Application, Yunnan Minzu University, No. 2929, Yuehua Street, Chenggong District, Kunming 650504, China; noname2306@ymu.edu.cn (M.Z.); 21227038010002@ymu.edu.cn (J.X.); r.zhang@ymu.edu.cn (R.Z.); tt041824@ymu.edu.cn (X.Y.); 041523@ymu.edu.cn (W.S.); 22227037640020@ymu.edu.cn (Z.Y.); phosphophyllite@ymu.edu.cn (Y.C.); 3125024229@stu.cpu.edu.cn (Y.T.); yuqiangzhao@ymu.edu.cn (Y.-Q.Z.); fanbm@ynni.edu.cn (B.F.); 2 Key Laboratory of Chemistry in Ethnic Medicinal Resources, State Ethnic Affairs Commission and Ministry of Education, Yunnan Minzu University, Kunming 650504, China

**Keywords:** *Bidens pilosa* L., tumor-associated macrophages, regulatory T cells, the tumor microenvironment, immunomodulator, chemical constituents

## Abstract

*Bidens pilosa* L., a traditional Chinese medicinal herb, has been used in clinical practice for the treatment of inflammatory diseases and cancer. **BPA**, an extract derived from the whole herb of *B. pilosa* L., has been shown to possess potent immunomodulatory properties by regulating tumor-associated macrophages (TAMs) and regulatory T cells (Tregs) within the tumor microenvironment (TME) in a mouse syngeneic colorectal cancer (CRC) model. RT-PCR and flow cytometry analyses showed that **BPA**, together with its flavonoid and polyacetylene constituents, effectively suppressed the differentiation of M2-TAMs and Tregs by downregulating Arg-1 and CD25 expression. They had minimal effects on the expression of markers associated with M1-TAMs and promoted the proliferation of CD4^+^ T cells that were inhibited by M2-TAMs and Tregs. In mice, **BPA** markedly inhibited the growth of syngeneic CRC tumors, accompanied by decreased serum levels of the immunosuppressive cytokine IL-10 and reduced expression of the proliferative marker Ki67 in tumor tissues. Moreover, **BPA** downregulated the mRNA expression of markers associated with M2-TAMs and Tregs, while increasing markers associated with M1-TAMs. Western blot analyses of tumor tissues revealed that **BPA** reduced the expression of marker proteins associated with M2-TAMs and Tregs, while increasing the expression of the immune-stimulatory markers CD80, GITR and CD4. In addition, combined treatment with **BPA** and 5-fluorouracil (5-FU), a commonly used chemotherapeutic agent for CRC, notably enhanced the anti-tumor effect in mice. These findings indicate that **BPA**, an active extract of *B. pilosa* L., showed antitumor activity in mice by suppressing the differentiation of pro-tumorigenic TAMs and Tregs within the TME.

## 1. Introduction

Cancer is one of the most prevalent diseases worldwide and ranks as the second leading cause of death among humans [1]. Although traditional cancer treatments, such as surgery, chemotherapy, and radiotherapy, have made significant progress, they are still associated with notable adverse effects and risks of recurrence and metastasis. Consequently, the development of novel therapeutic strategies to combat cancer is imperative [2]. In recent years, immunotherapy has emerged as a promising anticancer strategy by mobilizing host immunity against malignancies [3], and the rapid development of tumor immunotherapy has not only provided cancer patients with new treatment options but has also highlighted the crucial role of the tumor microenvironment (TME) in the processes of tumor initiation and progression [4].

The TME refers to the microenvironment surrounding tumor cells, which includes the tumor cells themselves, adjacent non-tumor cells, extracellular matrix, blood vessels, immune cells, as well as various signaling molecules and cytokines. Within the TME, immunosuppressive cells such as TAMs, Tregs, and MDSCs activate protumor signaling cascades that promote tumor immune evasion and drive disease pathogenesis [4,5]. As the most plastic immune cells abundant in the TME, TAMs can differentiate into either M1 or M2 macrophages. M1 macrophages, activated by stimuli such as interferon-γ (IFN-γ), release pro-inflammatory cytokines (e.g., interleukin-1β, IL-1β) and chemokines (such as CXCL10). This enhances antigen presentation, promotes Th1 responses, and mediates anti-tumor effects [6]. In contrast, M2 macrophages, activated by IL-4 and IL-13, secrete anti-inflammatory cytokines (e.g., IL-10 and TGF-β) that facilitate tumor development by promoting growth, angiogenesis, and the differentiation of CD4^+^ T cells into Tregs [7,8,9]. Tregs, characterized by the expression of Foxp3, play a crucial role in establishing immune tolerance within the TME by suppressing the activity of CD4^+^ and CD8^+^ T cells. They achieve this through the secretion of cytokines such as IL-10 and TGF-β, as well as by decreasing the expression of CD80 or CD86 on antigen-presenting cells, thereby facilitating immune evasion [10]. Overall, TAMs and Tregs play pivotal roles in creating the immunosuppressive TME, exerting powerful effects on tumor progression and clinical outcomes [11]. Therapeutic interventions targeting immunosuppressive TAMs and Tregs within the TME show clinical promise. For instance, emactuzumab, a monoclonal antibody targeting CSF-1R, effectively depletes M2 macrophages in tenosynovial giant cell tumors (TGCT), thereby restoring T cell activity and reducing tumor burden [12]. Sunitinib, a chemotherapy agent and tyrosine kinase inhibitor (TKI), selectively reduces the abundance and function of Tregs in patients with renal cell carcinoma, thereby enhancing anti-tumor immunity and therapeutic efficacy [13].

Natural products have attracted considerable attention for their capacity to modulate immune cell functions within the TME and to provide a promising strategy for cancer immunotherapy. Traditional Chinese medicine has a long history of clinical use for therapeutic purposes. *Bidens pilosa* L., an annual herb from the Asteraceae family, is utilized as both a food source and a traditional medicine for humans and animals. Traditional records highlight its application in treating a variety of diseases, particularly inflammatory diseases and cancer [14,15]. A diverse array of bioactive compounds has been identified from *B. pilosa* L. by phytochemical investigations, including flavonoids, polyacetylene, phenolic acids, terpenoids, lipids, and alkaloids. These constituents confer a wide range of pharmacological effects, including anti-hyperglycemic, antihypertensive, anti-ulcer, antipyretic, analgesic, immunosuppressive, antibacterial, anti-inflammatory activities, as well as antioxidant and antitumor effects [16]. The anticancer activity of *B. pilosa* L. is well-documented; however, its specific immunomodulatory effects in the context of cancer are not yet fully understood.

In our previous work, we isolated compounds from *B. pilosa* L. and characterized their structures. Preliminary studies demonstrated that flavonoids and polyacetylenes isolated from *B. pilosa* L. notably suppressed cancer cell proliferation by inhibiting DNA topoisomerase I (Topo I) and disrupting mitotic progression [17,18]. However, the immunomodulatory potential of these compounds within the TME, particularly their effect on immunosuppressive cells, remains largely unexplored. Therefore, this study focuses on TAMs and Tregs to investigate how **BPA**, an extract derived from *B. pilosa* L., exerts antitumor effects by modulating these immune cells within the mouse TME.

## 2. Materials and Methods

### 2.1. Plant Origin

In June 2020, *B. pilosa* L. was collected from Liangwang Mountain in Kunming, China and subsequently identified by Jun Zhang of Kunming Plant Science and Biotechnology Co., Ltd. (Kunming, China). The voucher specimen (YMU-ZF20200624) has been stored in the Yunnan Key Laboratory of Chiral Functional Substances Research and Application, Yunnan Minzu University.

### 2.2. Preparation of B. pilosa L. Extract

**Figure 1:**
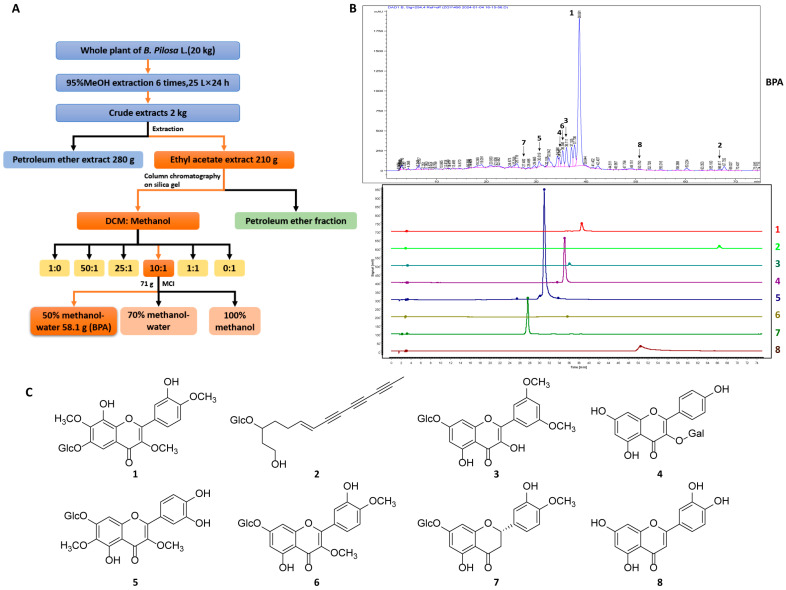
Extraction, HPLC analysis, and structural characterization of **BPA** and its main constituents. (**A**) Extraction and isolation process of **BPA**. (**B**) HPLC trace of **BPA**. HPLC analysis of **BPA** was performed using an Agilent 1260 Infinity II HPLC system with an Agilent XDB-C18 chromatographic column (dimensions: 4.6 × 250 mm, particle size: 5 μm) under gradient elution conditions with methanol. The specific methanol gradient profile was: 0–5 min, 10–20%; 5–15 min, 20–30%; 15–20 min, 30–35%; 20–30 min, 35–40%; 30–60 min, 40–45%; 60–70 min, 45–50%; 70–75 min and 50–55%. The effluents were monitored at 254 nm with the column maintained at 30 °C and a flow rate of 1.0 mL/min. (**C**) Chemical structures of compounds **1**–**8**, the major constituents of **BPA**.

Powdered whole plant of *B. pilosa* L. (20 kg) was cold-soaked in 95% methanol (MeOH) (60 L) for 24 h to obtain a filtrate, and the residue was then subjected to six additional extractions using 25 L of solvent each time. The crude extract weighing 2 kg was obtained by decompression concentration. Following the addition of 2 L of water, the crude extract was separated by petroleum ether (PE) and ethyl acetate (EtOAc). The EtOAc-solute fraction (210 g) was concentrated and fractionated on a silica gel column (60–100 mesh) (Qindao Marine Chemical Inc., Qingdao, China) followed by sequential elution using PE (35 g) and dichloromethane (DCM)-MeOH mixtures at volume ratios of (1:0 (11 g), 50:1 (28 g), 25:1 (9 g), 10:1 (81 g), 1:1 (17 g), and 0:1 (5 g)). The 10:1 DCM-MeOH fraction was concentrated and further purified by using MCI (Middle Chromatogram Isolated) gel (Mitsubishi Chemical, Japan) with 50% MeOH-50% H_2_O, yielding *B. pilosa* L. extract (**BPA**, 58.1 g) (Figure 1A). Compounds **1**–**8** were isolated from **BPA** and identified in HPLC trace of **BPA**. Additional details of the compounds are provided in Table S1.

### 2.3. Cell Culture

CT26. WT cells (colorectal cancer, murine) were obtained commercially (Guangzhou Cellcook Biotech Co., Ltd., Guangzhou, China) and cultured in Dulbecco’s Modified Eagle Medium (DMEM, Biological Industries, Cat. C3113-0500, Beit Shemesh, Israel) supplemented with 10% (*v*/*v*) FBS (VivaCell, Cat. C04001-500, Shanghai, China) and 1% glutamine (Biological Industries, Cat. E607004-0500) under standard conditions (37 °C, 5% CO_2_) in a humidified incubator (ThermoFisher, Waltham, MA, USA).

### 2.4. Animal Experiments

Male BALB/c or Kunming mice aged 6–8 weeks were purchased commercially (Henan Skobes Biotechnology Co., Ltd., Anyang, China) and maintained in specific pathogen-free (SPF) housing with controlled environmental parameters (temperature: 23 ± 2 °C; humidity: 55 ± 10%; 12 h light/dark). The animal Experiment Ethics Committee of Yunnan Minzu University approved all protocols (Issue No. YMU-2022-A027; YMU-AFEC-2023-A001; YMU-AFEC-2023-A009).

#### 2.4.1. Isolation and Polarization of Peritoneal Macrophages

Peritoneal macrophages were isolated from Kunming mice by peritoneal lavage according to the method described by Zhao et al. [19], and then resuspended in RPMI 1640 medium (Biological Industries, Cat. C3010-0500) supplemented with 10% (*v*/*v*) FBS, 1% Penicillin-streptomycin (Sangon Biotech, Cat. E607011-0100, Shanghai, China). After 3 h for adherence, non-adherent cells were removed to yield peritoneal macrophages (M0). M0 macrophages were treated with 20 ng/mL of IL-4 (PeproTech, Cat. 214-14, Cranbury, NJ, USA) and 20 ng/mL of IL-13 (Sino Biological Inc., Cat. 50225-MNAH, Beijing, China) for 72 h to facilitate their transformation into M2 macrophages, or were treated with 1 μg/mL LPS (Sigma, Cat. 93572-42-0, St. Louis, MO, USA) for 24 h to promote their transformation into M1 macrophages.

#### 2.4.2. Isolation and Differentiation of Induced Regulatory T Cells

Lymphocytes were isolated from the lymph nodes and spleens of Kunming mice as previously described [20]. The resulting cell suspension was passed through a 70-μm cell strainer (NEST, Cat. 258368, Wuxi, China), followed by centrifugation and resuspension in EasySep buffer to a final concentration of 1 × 10^8^ cells/mL, then naïve CD4^+^ T cells were purified from this suspension using the EasySep mouse naïve CD4^+^ T cell isolation kit (Stemcell Technologies, Cat. 19765, Vancouver, BC, Canada) according to the manufacturer’s instructions. The purity of isolated CD4^+^ T cells was assessed by flow cytometry, and results are presented in Figure S2B. To obtain induced Tregs, 24-well plates were pre-coated with anti-CD3e (5 μg/mL, eBioscience, Cat. 16-0031-82, San Diego, CA, USA) overnight at 4 °C. Purified naïve CD4^+^ T cells were then added at 2 × 10^6^ cells/well in Treg-polarizing medium supplemented with anti-CD28 (2 μg/mL, eBioscience, Cat. 16-0281-82), anti-IL-4 monoclonal antibody (5 μg/mL, eBioscience, Cat. 16-7041-81), anti-IFN-γ antibody (5 μg/mL, eBioscience, Cat. 16-7311-85), IL-2 (100 U/mL, Sino Biological Inc., Cat. 51061-MNAE, Beijing, China), TGF-β (4 ng/mL, Sino Biological Inc., Cat. 51061-MNAE), and rapamycin (100 ng/mL, Sigma, Cat. S115842). After that, cells were incubated at 37 °C for 96 h to induce into Tregs.

#### 2.4.3. Syngeneic Tumor Model in Mice

CT26. WT cells in logarithmic growth phase were harvested, washed, and resuspended in DMEM at a concentration of 8 × 10^6^ cells/mL and an aliquot of cell suspension (0.1 mL) was injected subcutaneously in the right flank of each BALB/c mouse to initiate tumor growth. Tumor length (a) and width (d) were measured with a vernier caliper, and tumor volume (V) was calculated as V = 0.5 × a × d^2^.

When subcutaneous tumors reached a volume of 50–100 mm^3^, mice were randomly assigned to five groups (*n* = 6 per group) using simple randomization. No significant differences in tumor volume or body weight were observed among the groups prior to treatment. The groups were defined as follows: The model group served as the negative control and received daily oral administration of 0.5% carboxymethylcellulose sodium (CMC-Na). The positive control group received 5-FU (MCE, Cat. HY-90006, Monmouth Junction, NJ, USA) at a dose of 30 mg/kg by intraperitoneal injection every three days. The **BPA** treatment groups were given daily oral **BPA** at 100 mg/kg or 50 mg/kg, each dissolved in 0.5% CMC-Na. The combination treatment group received 5-FU by intraperitoneal injections at 30 mg/kg every 3 days, together with daily **BPA** gavage at 50 mg/kg. Animals had free access to water and food throughout the study. Once tumor volumes reached approximately 1500 mm^3^, mice were euthanized, and serum and tissue specimens, including liver, heart, spleen, lungs, kidneys, stomach, and tumors, were collected for subsequent analyses. Normal BALB/c mice that did not undergo subcutaneous CT26. WT cell injections were used as sham controls. The investigators performing outcome assessments and the personnel administering treatments were blinded to group allocation.

### 2.5. MTT Assay

For M2-TAMs, M0 macrophages were seeded in 96-well plates at a density of 5 × 10^4^ cells/well, followed by the addition of IL-4 and IL-13. After 24 h of stimulation, different concentration of **BPA** or other compounds were added and then incubated for 48 h. Cell viability was determined by MTT assay (Macklin, Cat. 298-93-1, Shanghai, China) and measured on a microplate reader (SpectraMax i3x, Molecular Devices, Sunnyvale, CA, USA). For Tregs, purified naïve CD4^+^ T cells were seeded at 5 × 10^5^ cells/well in 96-well plates precoated with anti-CD3e. Corresponding stimulatory factors and test samples were then added. After 96 h of incubation, cells were stained with 0.04% trypan blue and viable cells were counted with a hemocytometer. IC_50_ values were calculated by the Reed&Muench method and are expressed as the mean ± SEM from at least three independent measurements, each performed in duplicate.

### 2.6. Flow Cytometry

M2 macrophages treated with or without samples were harvested and resuspended in PBS. To block Fc receptors, cells were incubated with anti-CD16/32 (Elabscience, Cat. E-AB-F0997A, Wuhan, China) for 10 min. After washing, cells were stained with PerCP/Cyanine 5.5 anti-mouse F4/80 (Elabscience, Cat. E-AB-F0995J), FITC anti-mouse CD206 antibody (Elabscience, Cat. E-AB-F1135C), and APC anti-mouse CD80 antibody (Elabscience, Cat. E-AB-F0992E) at 4 °C for 30 min. The population of CD206^+^ or CD80^+^ were analyzed in F4/80^+^ cells using a Beckman CytoFlex flow cytometry (Figure S2). Induced Tregs treated with or without samples were resuspended in PBS and stained with APC anti-mouse CD25 antibody (Elabscience, Cat. E-AB-F1102E) for 30 min at 4 °C. Cells were then fixed and permeabilized using the Transcription Factor Buffer Set kit (BD Biosciences, Cat. 562574, Franklin Lakes, NJ, USA) according to the manufacture’s instruction. After incubated with PE anti-mouse Foxp3 antibody (Elabscience, Cat. E-AB-F1238D) at 4 °C for 50 min, cells were analyzed by flow cytometry for CD25^+^Foxp3^+^ cell populations. Flow cytometry analysis results are based on at least three independent experiments.

### 2.7. Quantitative Real-Time PCR (qRT-PCR)

The total RNA of M1/M2 macrophages, Tregs and tumor tissues was extracted using a Total RNA Extractor (Trizol) Extraction Kit (TaKaRa, Cat. 9109, Kusatsu, Japan) and reverse-transcribed into cDNA with a reverse transcription kit (Vazyme, Cat. R223-01, Nanjing, China). The cDNA was used for PCR amplification in accordance with the method described in previous publication [21]. Relative gene expression levels were normalized to 18S rRNA and calculated via 2^−ΔΔCt^ method. Quantitative RT-PCR results are from at least three independent experiments. Primer sequences used in this study are listed in Supplementary Table S2.

### 2.8. T Cell Proliferation Assay

Purified CD4^+^ T cells were labeled with 0.5 μM CFSE (MCE, Cat. HY-D0938) in PBS for 10 min at 37 °C, followed by washing and resuspension in complete RPMI-1640 medium. The cells were seeded in 96-well plates precoated with anti-CD3e and co-cultured with M2 macrophages treated with or without **BPA** at a 5:1 ratio (T cells: M2 macrophages), or with Tregs treated with or without **BPA** at a 2:1 ratio (T cells: Tregs), in the presence of anti-CD28 mAb. After 72 h incubation, cells were collected, resuspended in staining buffer, and the proliferation of CFSE-labeled CD4^+^ T cells was then assessed by flow cytometry. The analysis results are based on at least three independent experiments.

### 2.9. ELISA

Whole blood was collected from mice and allowed to clot at room temperature for 3 h. Serum was obtained by centrifugation. Serum IL-10 levels were detected using a mouse IL-10 ELISA Kit (Proteintech, Cat. KE10008, Wuhan, China) according to the manufacturer’s instructions.

### 2.10. Hematoxylin/Eosin Staining

Tissues from mice (tumor, heart, liver, spleen, lung, kidney, and stomach) were fixed in 10% neutral-buffered formalin for 48 h, dehydrated, embedded in paraffin, and sectioned. Sections were stained with hematoxylin and eosin (H&E) using commercial kits (Solarbio, Cat. G1140/G1100, Beijing, China) according to the manufacturer’s instructions. After mounting with neutral resin (Solarbio, Cat. G8590), images were captured with an inverted microscope (Leica DMi8, Wetzlar, Germany).

### 2.11. Immunohistochemical

Tumor sections were rehydrated and subjected to antigen retrieval in Tris-EDTA buffer (10 mM Tris, 1 mM EDTA, 0.05% Tween-20, pH 8.0) using an autoclave for 2 min. After washing, sections were blocked with 10% goat serum (Solarbio, Cat. SL038) in TBS containing 1% BSA (Coolaber, Cat. CA1381, Beijing, China) at 37 °C for 1 h, then incubated overnight at 4 °C with anti-Ki67 primary antibody (Proteintech, Cat. 28074-1-AP). A horseradish peroxidase-polymer-conjugated secondary antibody (ZSGB-BIO, Cat. PV-6001, Beijing, China) was applied at 37 °C for 30 min, followed by DAB detection (ZSGB-BIO, Cat. ZLI-9018) and hematoxylin counterstain. Slides were mounted and imaged using an inverted microscope.

### 2.12. Western Blot

Approximately 10 mg of tumor tissue was weighted and added to lysis buffer (2% SDS, 10% glycerol, 65 mM Tris-HCl, pH 6.8), then homogenized on ice using a tissue grinder. Lysate was sonicated and subsequently heated at 98 °C for 10 min. Further methodological details were performed as previously described [21]. Band densities were quantified by grayscale analysis in ImageJ software (version 1.53a) from at least three independent experiments. Antibody details used in this study is presented in Supplementary Table S3.

### 2.13. RNA Sequencing (RNA-Seq) and Bioinformatic Analysis

Total RNA was extracted from TAMs and Tregs treated with stimulatory factors and different concentrations of **BPA** using TRIzol reagent. RNA samples that met quality control criteria were used to construct sequencing libraries with the NEB library preparation protocol. Library insert size distributions were validated on an Agilent 2100 Bioanalyzer. Qualified libraries were quantified and sequenced on an Illumina platform (Novogene, Beijing, China).

For RNA-seq data analysis, sequencing quality was assessed from base-calling output generated by CASAVA. Sequenced reads were mapped to GRCm39 genome using hisat2 software (version 2.2.1), and gene-level read counts were generated with featureCounts. After normalization, differential gene expression analysis was carried out using the R package DESeq2 (version 4.5.1). Volcano plot analysis was performed with the R package ggplot2 (version 4.5.1) to identify differentially expressed genes (DEGs) with a *p* ≤ 0.05 and an absolute value of log_2_ fold change ≥ 1. Functional enrichment analysis of DEGs was conducted using the R package clusterProfiler (version 4.5.1) for Gene Ontology (GO) and Kyoto Encyclopedia of Genes and Genomes (KEGG) pathway annotation.

### 2.14. Statistical Analysis

Quantitative data are expressed as mean ± SEM from at least three independent experiments. Statistical significance was determined using either one-way ANOVA or Student’s *t*-test in GraphPad Prism (version 8.0.2). A *p* value threshold of less than 0.05 was applied to define statistical significance.

## 3. Results

### 3.1. The Chemical Characterization of BPA

The chemical profile of **BPA** was characterized using HPLC through comparison with compounds isolated from *B. pilosa* L. Detailed extraction and compounds identification were described in our previous studies [18,19]. Seven flavonoids (**1**, **3**–**8**) and one polyacetylene (**2**) were identified in the HPLC trace of **BPA** (Figure 1B,C), in which **1** and **3** are the main components. Both **BPA** and its chemical components (**1**–**8**) were subsequently used in vitro to assess their modulatory effects on TAMs and Tregs.

### 3.2. BPA and **1–8** Do Not Show Potent Cytotoxicity on M2 Macrophages and Tregs

**Figure 2:**
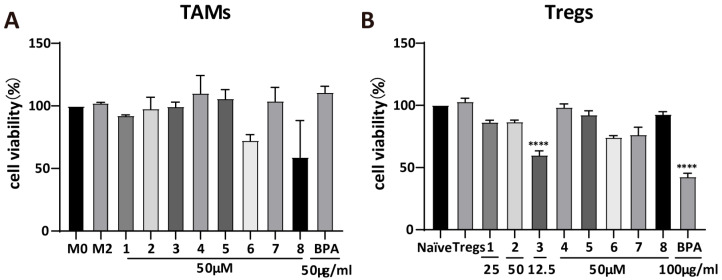
Cytotoxicity of **BPA** and compounds **1**–**8**. Cytotoxic effects of **BPA** and **1**–**8** on M2 macrophages (**A**) and Tregs (**B**). Statistical significance relative to the M2 macrophage or Tregs group is indicated as follows (*n* = 3): **** *p* < 0.0001.

M0 macrophages and naïve CD4^+^ T cells were differentiated into M2 macrophages and Tregs, respectively, and subsequently treated with the appropriate concentrations of **BPA** and compounds **1**–**8**. Cytotoxicity assay revealed that, at 50 μM, most compounds had minimal inhibitory effects on M2 macrophage viability (Figure 2A). However, compounds **6** and **8** represented exceptions, reducing cell viability by approximately 30% and 40%, respectively. For Tregs (Figure 2B), most compounds reduced cell viability by approximately 10%, whereas **3** decreased it by roughly 30%. At the concentration of 50 μg/mL, **BPA** markedly suppressed the cell viability of Tregs by 55%. These results indicate that **BPA** and its monomeric compounds showed a weak inhibitory effect on both M2 macrophages and Tregs.

### 3.3. BPA Inhibits the Differentiation of M0 Macrophages into M2 Macrophages and the Polarization of CD4^+^ T Cells into Tregs

**Figure 3:**
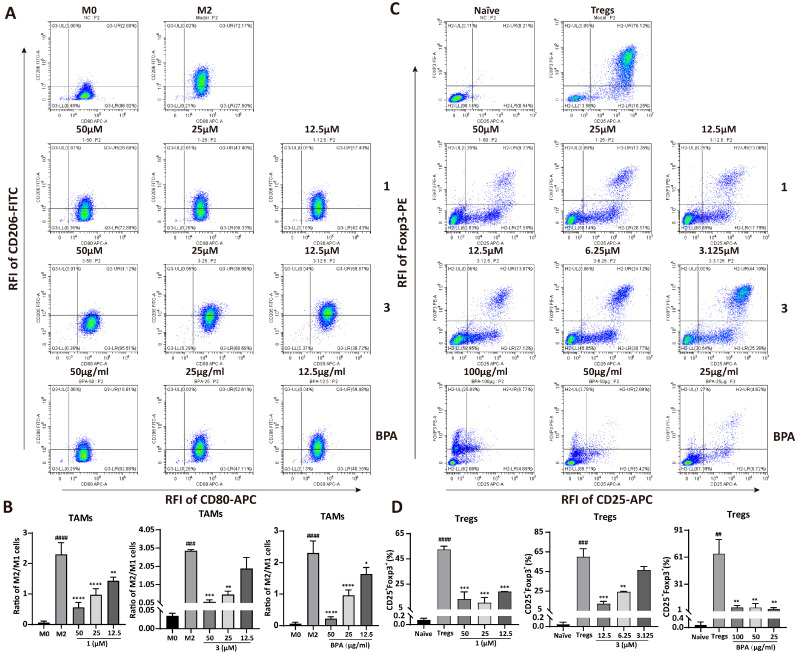
Effect of **BPA** and compounds **1** and **3** on the differentiation of macrophages and CD4^+^ T cells. (**A**) Representative flow cytometry plots illustrating the effect of **BPA**, **1**, and **3** on the differentiation of M2 macrophages. (**B**) Quantitative analysis of the ratios of M2 to M1 cells following treatment with various concentrations of **BPA**, **1** and **3**. (**C**) Representative flow cytometry plots illustrating the effect of **BPA**, **1**, and **3** on the differentiation of Tregs. (**D**) Histograms displaying the quantified results of the CD25^+^Foxp3^+^ double-positive cell population following treatment with different concentrations of **BPA**, **1** and **3**. Statistical significance for all experiments is denoted as follows (*n* = 3): compared to the M0 cells or Naïve CD4^+^ T cells, ^##^ *p* < 0.01, ^###^ *p* < 0.001, ^####^ *p* < 0.0001; compared to M2-TAMs or Tregs, * *p* < 0.05, ** *p* < 0.01, *** *p* < 0.001, and **** *p* < 0.0001. RFI means Relative Fluorescence Intensity.

TAMs and Tregs promote tumor progression through the establishment of immunosuppressive microenvironments. To assess the effects of **BPA** and its chemical constituents (**1**–**8**) on immune cell differentiation, specifically the conversion of M0 macrophages into either M1 or M2 macrophages, as well as the differentiation of T cells into Tregs, we performed qRT-PCR and flow cytometry analyses. Results from qRT-PCR revealed that LPS significantly enhanced the mRNA expression levels of iNOS (*p* = 0.026) and IL-1β (*p* = 0.0397), which are markers of M1 macrophages (Figure S1C,D). By contrast, **BPA** and compounds **1** and **3**–**8** did not induce significant changes in the expression of these transcripts relative to the M1 control (Figure S1C), indicating that they do not affect M1 differentiation. Results from flow cytometry confirmed this finding, showing no obvious changes in CD80^+^F4/80^+^ cell populations (M1 macrophages) after treatment (Figure S3E,F). However, almost all compounds and **BPA** notably decreased the mRNA expression of the M2 macrophage marker Arg-1 (Figure S1A) and the regulatory T cell marker CD25 (Figure S1B). Consistent with these findings, flow cytometric analysis demonstrated a pronounced reduction in immunosuppressive cell populations, including M2 macrophages (CD206^+^F4/80^+^) and Tregs (Foxp3^+^CD25^+^), upon treatment (Figure S3A–D). Furthermore, **BPA** and **1**–**8** significantly reduced the CD206^+^/CD80^+^ cell ratio in F4/80^+^ macrophages (*p* = 4.4 × 10^−7^, Figure S3G,H). Compounds **1** and **3**, identified as the major constituents of **BPA** [18], were studied along with **BPA** in subsequent investigation. Flow cytometric analysis revealed that the population of M2 macrophages (CD206^+^F4/80^+^) showed dose-dependent reductions (Figure S4C,D) following treatment with **BPA**, **1**, and **3**. In contrast, the CD80^+^F4/80^+^ cell populations (M1 macrophages) remained unchanged (Figure S4A,B), suggesting a selective effect on M2 macrophages. Additionally, the CD206^+^/CD80^+^ cell ratio declined in a concentration-dependent manner with increasing concentrations of **1**, **3**, and **BPA** (Figure 3A,B). Notably, only compound **3** induced a dose-dependent suppression of Tregs. In contrast, **1** and **BPA** showed potent inhibitory effects on differentiation of Tregs, although not in a concentration-dependent manner (Figure 3C,D). This lack of concentration dependence may be due to the drug’s effects on Tregs cytotoxicity and other complex underlying mechanisms. Collectively, these data indicate that **BPA** exerts an in vitro immunomodulatory effect by inhibiting the proliferation of both M2-TAMs and Tregs.

### 3.4. BPA Reverses M2 Macrophage- and Treg-Mediated Suppression of CD4^+^ T Cells

**Figure 4:**
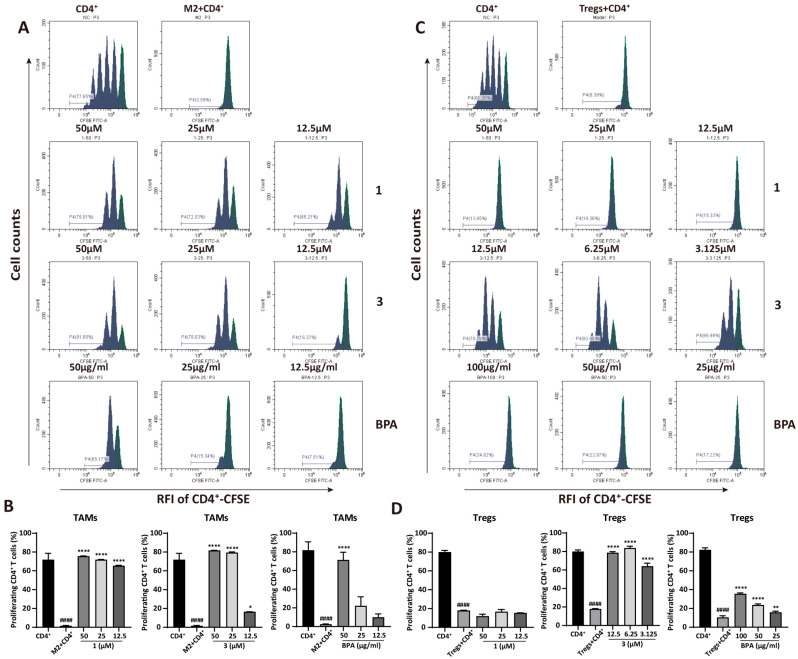
**BPA** and compounds **1** and **3** enhance CD4^+^ T cell proliferation suppressed by M2 macrophages and Tregs. (**A**) Representative flow cytometry plots illustrating the effect of **BPA**, **1**, and **3** on CD4^+^ T cell proliferation suppressed by M2 macrophages. (**B**) Quantification of CD4^+^ T cell proliferation following co-culture with M2 macrophages pretreated with **BPA**, **1** and **3**. (**C**) Representative flow cytometry plots illustrating the effect of **BPA**, compounds **1**, and **3** on CD4^+^ T cell proliferation inhibited by Tregs. (**D**) Quantification of CD4^+^ T cell proliferation following co-culture with Tregs pretreated with **BPA**, **1** and **3**. Statistical significance is indicated as follows (*n* = 3): compared to the CD4^+^ group, ^####^ *p* < 0.0001; Compared to the M2 or Tregs group, * *p* < 0.05, ** *p* < 0.01, **** *p* < 0.0001. RFI means Relative Fluorescence Intensity.

The anti-tumor immune response is compromised when M2 macrophages and Tregs suppress Th1 cytokine secretion, directly inhibiting the proliferation and activation of CD4^+^ T cells. This suppression subsequently promotes tumor progression. To determine whether **1**, **3**, and **BPA** can reverse this suppression, CD4^+^ T cell proliferation in co-culture was measured by CFSE staining and analyzed by flow cytometry. Results from Figure 4 exhibited that both M2 macrophages and Tregs significantly inhibited CD4^+^ T cell proliferation, with the proliferation rate decreased from 77.65% to 2.59% (*p* = 5.45 × 10^−6^) and 81.20% to 8.39% (*p* = 5.52 × 10^−10^), respectively. However, a concentration-dependent increase in CD4^+^ T cell proliferation was observed following pretreatment of M2 macrophages with **1**, **3**, or **BPA** (Figure 4A,B). In particular, compound **1** at the concentrations of 50, 25, and 12.5 μM markedly increased CD4^+^ T cells proliferation to 75.81%, 72.03%, and 65.21%, respectively (*p* = 0.000016, *p* = 0.000019, and *p* = 0.000026), compared to the M2 macrophage-suppressed CD4^+^ T cell group. As shown in Figure 4C,D, pretreatment of Tregs with compound **3** at 12.5, 6.25, and 3.125 μM significantly enhanced CD4^+^ T-cell proliferation from 8.39% to 79.79%, 82.65%, and 66.99%, respectively (*p* = 7.29 × 10^−6^, *p* = 4.97 × 10^−6^, and *p* = 0.000019). **BPA** showed a similar but less pronounced effect, whereas compound **1** did not cause statistically significant changes in regulatory T cell-mediated suppression. Therefore, **BPA** may attenuate the immunosuppressive activity of M2 macrophages and Tregs, leading to restoration of CD4^+^ T cell proliferation and a potential enhancement of antitumor immune response.

### 3.5. BPA Inhibits the Growth of Syngeneic Colorectal Cancer in Mice

**Figure 5:**
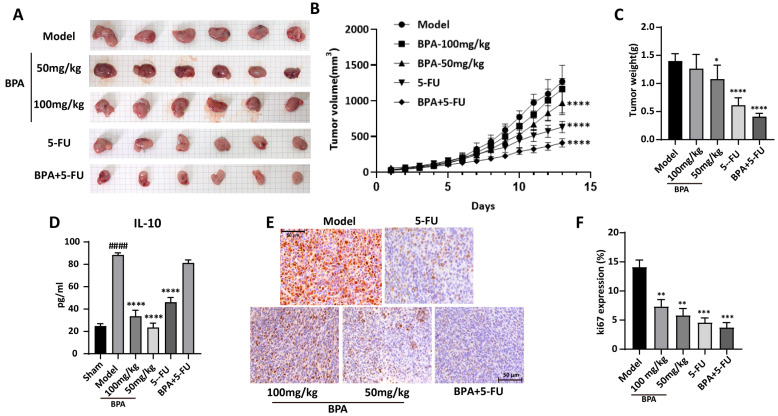
The growth of CT26. WT syngeneic tumors in mice is inhibited by **BPA**. (**A**) Images of CT26. WT syngeneic tumors in mice from different treatment groups. Scale bar = 5 mm (each background grid square corresponds to 5 mm). (**B**) Tumor growth curves for syngeneic CT26.WT tumors in mice were generated from daily volume measurements during the treatment phase (n = 6). (**C**) Statistical analysis of tumor weight at the end of the study (n = 6). (**D**) Statistical analysis of serum IL-10 levels (*n* = 6). (**E**) Representative immunohistochemistry (IHC) images showing the expression of the Ki67 protein in tumor tissue sections from different treatment groups. Original magnification 400×. (**F**) Quantification of Ki-67 expression in tumor tissue. The proportion of Ki-67-positive area within sections was quantified using ImageJ software and is presented as a percentage of total tissue area. Statistical significance is denoted as follows: compared to the sham group, ^####^ *p* < 0.0001; Compared to the model group, * *p* < 0.05, ** *p* < 0.01, *** *p* < 0.001, **** *p* < 0.0001.

Tumor growth rate and volume are key indicators of tumor progression. To validate the in vitro findings, a murine syngeneic tumor model was established using CT26. WT cells to assess the in vivo effects of **BPA** on tumor growth. Compared to model controls, **BPA** treatment suppressed tumor growth and reduced tumor weight (Figure 5A–C). Additionally, combined treatment with **BPA** and 5-FU exhibited stronger antitumor effect compared to 5-FU monotherapy, which served as the positive control. Notably, no significant body weight changes were observed across groups, suggesting that **BPA** has minimal systemic toxicity in mice (Figure S5A). H&E histopathological analysis also showed no obvious pathological changes in major mouse organs (heart, liver, spleen, lungs, kidneys, and stomach) compared to healthy controls (Figure S5B). These findings suggest that **BPA** inhibits colorectal cancer growth in mice without causing significant toxicity or organ damage.

IL-10, a key immunosuppressive cytokine secreted by TAMs and Tregs, is essential for maintaining an immunosuppressive tumor microenvironment. ELISA quantification of Serum IL-10 revealed a significant increase in the model group (*p* = 6.71 × 10^−10^), which was substantially attenuated following **BPA** treatment (Figure 5D). Although the 5-FU and the combination treatments led to a reduction in IL-10 levels, these decreases were less pronounced compared to the effects observed with **BPA** alone. The anti-proliferative effect of **BPA** on tumor cells was investigated by immunohistochemical detection of Ki67, a key marker of cell proliferation. Notably, Ki67 expression was significantly decreased in the **BPA**-treated group compared to the model group (Figure 5E,F), consistent with the observed reduction in tumor volume and weight. Taken together, these results indicate the antitumor effect of **BPA**, either used alone or with conventional chemotherapy (e.g., 5-FU), for the treatment of colorectal cancer.

### 3.6. BPA Modulates the Expression of Genes and Proteins Associated with TAMs and Tregs in Mouse Tumor Tissues

**Figure 6:**
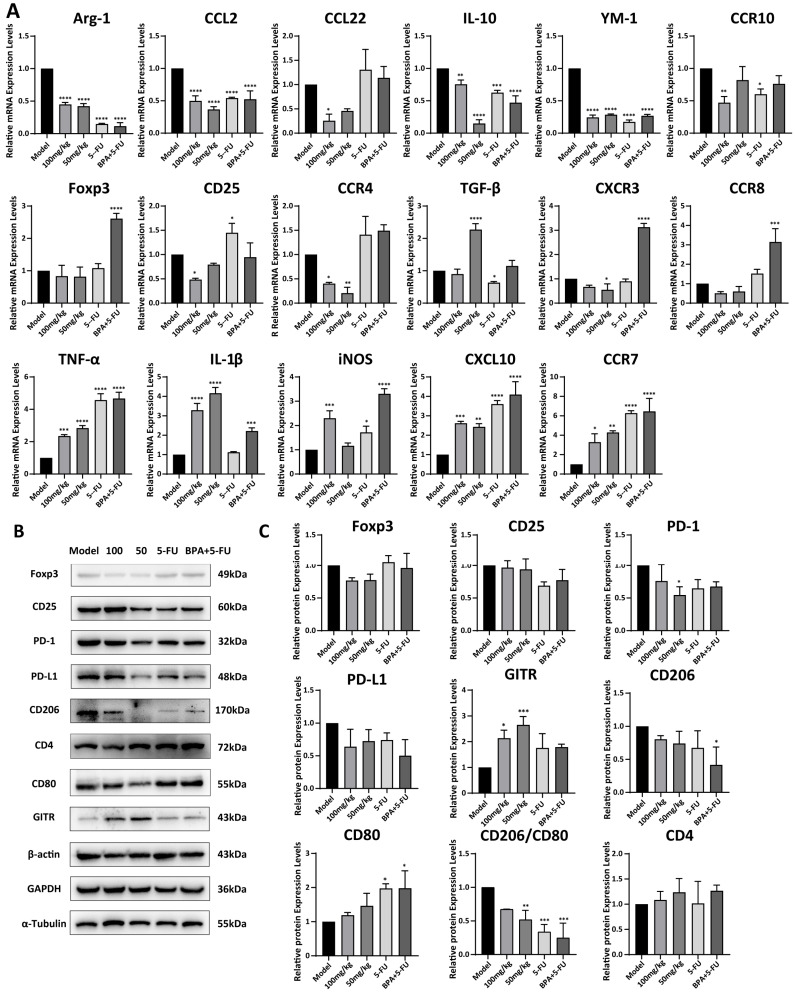
Expression levels of mRNAs and proteins associated with TAMs and Tregs in **BPA**-treated mouse tumors. (**A**) Quantification of mRNA by qRT-PCR for TAM/Treg-associated tumor-promoting factors (Arg-1, YM-1, CCL2, CCL22, Foxp3, CD25, TGF-β, CXCR3, CCR4, CCR8, CCR10) and tumor-inhibitory factors (TNF-α, IL-1β, iNOS, CXCL10, CCR7). Data were normalized to 18 S rRNA and are shown relative to the model group (baseline control). (**B**) Representative immunoblots of tumor tissue for TAM- and Treg-associated proteins. Pro-tumor: CD206, Foxp3, CD25, PD-1, PD-L1; Anti-tumor: CD80, CD4, GITR. (**C**) Quantification of protein expression from Western blots. Values were normalized to α-tubulin, β-actin, or GAPDH as indicated. Statistical significance is denoted as follows (*n* = 6): compared to the model group, * *p* < 0.05, ** *p* < 0.01, *** *p* < 0.001, **** *p* < 0.0001.

The expression levels of genes in mouse tumor tissues were analyzed by qRT-PCR, and the results demonstrated that treatment with 100 mg/kg **BPA** significantly reduced the mRNA levels of the tumor-promoting factors, including Arg-1, YM-1, CCL2, CCL22, CD25, CCR4, and CCR10, secreted by TAMs and Tregs, compared to the model group, respectively (all *p* < 0.01). Whereas, the levels of TGF-β, CXCR3, and CCR8 showed only a slight reduction. This indicates that **BPA** might transform the TME from immunosuppressive to immune-supportive by modulating cytokine or chemokine levels within the tumor. Moreover, **BPA** also increased the mRNA levels of the tumor-inhibitory factors, including TNF-α, IL-1β, iNOS, CXCL10, and CCR7, compared to the model (Figure 6A). These findings indicates that **BPA** might promote a transition of the tumor microenvironment toward immune supportiveness by modulating cytokine and chemokine levels. The expression levels of proteins in tumors were analyzed by Western blot (Figure 6B,C), and the results exhibited that treatment with **BPA** led to a reduction in the expression levels of CD206 and PD-1. In contrast, there were no significant changes observed in the levels of CD25, CD80, and PD-L1. Notably, there was a significant increase in levels of GITR (100 mg/kg: *p* = 0.01; 50 mg/kg: *p* = 0.00069). Additionally, the ratio of CD206 to CD80 protein levels, which serves an indicator of the functional state of immune cells, particularly macrophages, was calculated and revealed a significant decrease in **BPA**-treated tumors compared to the model group (100 mg/kg: *p* = 0.062; 50 mg/kg: *p* = 0.0061). To investigate whether the immunoregulatory effect of **BPA** can enhance the anti-tumor effects of chemotherapy drugs, we established a combination treatment group of **BPA** and 5-FU, a clinically used drug for CRC chemotherapy. The results showed that the combination therapy significantly reduced the CD206/CD80 protein ratio (*p* = 0.0002) in tumor tissues (Figure 6B,C). These results indicate that **BPA** reduced the prevalence of M2 macrophages within the TME, either alone or in combination with 5-FU.

### 3.7. BPA Downregulates Pro-Tumor Pathways in TAMs and Tregs

**Figure 7:**
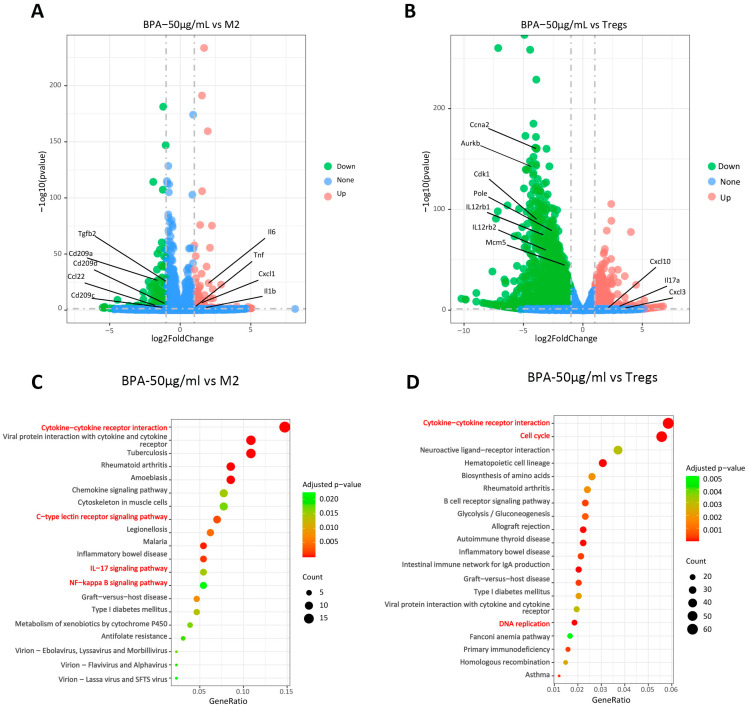
**BPA** inhibits the expression of tumor-promoting related pathways in TAMs and Tregs. (**A**) Volcano plot of RNA-seq results for TAMs; (**B**) Volcano plot of RNA-seq results for Tregs, where red dots represent upregulated genes and blue dots represent downregulated genes. (**C**) Scatter plot of KEGG-enriched pathways from the biological process analysis of TAMs; (**D**) Scatter plot of KEGG-enriched pathways from the biological process analysis of Tregs. The signaling pathways highlighted in red are closely related to the effects of **BPA**.

Collectively, our results indicate that **BPA** inhibits the differentiation of TAMs toward the pro-tumor M2-like TAMs and of Naïve CD4^+^ T cells toward pro-tumor Tregs within the TME. However, the precise molecular pathways by which **BPA** modulates TAMs and Tregs remain unclear. To further elucidate the regulatory mechanisms, we induced M2 macrophages and Tregs in vitro in the presence of **BPA** and performed transcriptome sequencing, and the representative findings are presented in Figure 7. A total of 296 differentially expressed genes (DEGs) were identified between the **BPA**-treated and M2-TAMs groups (Figure 7A). Among these, genes encoding immunosuppressive factors (TGF-β2, CD28, Hgf) were significantly downregulated, whereas genes associated with inflammation and immune activation (Il-6, TNF, CXCL1, IL-1β) were markedly upregulated (Figure 7B). This coordinated dysregulation of these genes is likely to contribute to **BPA**-mediated inhibition of the differentiation of M0 macrophages into M2 macrophages. KEGG pathway enrichment analysis revealed significant alterations in the IL-17, NF-κB, and C-type lectin receptor signaling pathways (Figure 7C), all of which play important roles in immunomodulation. Compared with the Tregs group, **BPA** treatment identified 2935 DEGs, including marked alteration of cytokine- and chemokine-related genes (IL-17a, Cxcl-10, Cxcl3, IL12rb1, IL12rb2, etc.) and downregulation of genes involved in cell proliferation and survival (Ccna2, Cdk1, Aurkb, Mcm5, Pole, etc.). These transcriptional changes may underlie **BPA**-mediated inhibition of regulatory T cell differentiation and proliferation. KEGG pathway enrichment analysis revealed significant perturbations in the cell cycle, DNA replication and cytokine-cytokine receptor interaction (Figure 7D). The first two pathways, cell cycle and DNA replication, are involved in development as well as cellular survival and proliferation. These perturbations are consistent with **BPA**-induced in Tregs observed in Figure 2B.

## 4. Discussion

Flavonoids have been isolated from various plants and demonstrated diverse biological activities, including tumor suppression, immunomodulation, and antioxidant effects [22]. The Chinese medicinal herb *B. pilosa* L., traditionally used in cancer treatment, contains a large number of flavonoids (>100) that exhibit antioxidant, anticancer, and other bioactive properties [23]. In our investigation of the anti-tumor activity of plant-derived compounds, we focused on the chemical constituents of *B. pilosa* L. and identified a total of 80 compounds, including 19 flavonoids and 10 polyacetynes. Notably, 13 flavonoids and 5 polyacetynes were specifically isolated from **BPA** [18,19]. Cytotoxicity and DNA topoisomerase I (Topo I) inhibition assays revealed that several flavonoids and polyacetynes exhibited potent cytotoxicity on a panel of 6 cancer cell lines by inhibiting the activity of Topo I. However, most of the compounds isolated from **BPA** did not exhibit inhibition on tumor cell growth or on Topo I activity [18]. Consequently, we further explored the immunomodulatory effects of **BPA** and its chemical constituents in the context of tumor treatment, with a particular focus on the TME.

Given the pivotal role of the TME in tumorigenesis, progression, and metastatic dissemination, immunotherapy represents a promising strategy for reshaping the TME and enhancing antitumor immune response [24]. Although targeting TAMs and Tregs within the TME shows considerable therapeutic potential, current agents, including those in clinical investigation, lack robust in vitro validation [4]. This shortcoming has resulted in the absence of appropriate positive controls targeting TAMs and Tregs in our experimental designs. HPLC quantification confirmed that compounds **1**–**8** are the predominant constituents of **BPA**, with compound **1** exhibiting the highest abundance (Figure 1). Cytotoxicity results revealed that compared to TAMs, **BPA** and **1**–**8** exhibited weak cytotoxic activity against Tregs, whereas no significant cytotoxicity was observed in TAMs, except compound **8** (Figure 2A,B).

As the anti-tumor immune cells, M1-TAMs express iNOS and produce IL-1β, and display the surface marker CD86 and CD80; by contrast, as the tumor promoting immune cells, M2-TAMs express Arg-1 and YM-1, secrete CCL2, end display CD163 and CD206. Tregs expresses CD25 and Foxp3 [25,26]. Results from RT-PCR (Figure S1) and flow cytometry (Figure S3) revealed that **BPA** and **1**–**8** modulated the differentiation of M0 macrophages into M2-type macrophages and inhibited the conversion of Naïve CD4^+^ T cells into Tregs. The populations of CD80^+^CD206^+^ in F4/80^+^ cells were analyzed (Figure S3C–H), along with the population of CD25^+^Foxp3^+^ in CD4^+^ T cells (Figure S3A,B). The results indicated that **BPA** and **1**–**8** more effectively inhibited M2 polarization and suppressed regulatory T cell differentiation, while exhibiting no significant activity on M1 polarization (Figure S3).

During the purification process, compound **1** and **3** were obtained as a mixture through crystallization. Consequently, further investigation primarily focused on **BPA** and its main flavonoids constituents, **1** and **3**. The results from flow cytometry analysis demonstrated that **1** and **3** inhibited the transformation of M0 macrophages and Naïve CD4^+^ T cells into their immunosuppressive forms in a concentration-dependent manner (Figure 3A–D). Specifically, **BPA** showed a concentration-dependent inhibition of the CD206^+^F4/80^+^ cell population (Figure S4C,D). Although **BPA** reduced the CD25^+^Foxp3^+^ cell population, this effect was not dose-dependent, likely due to its cytotoxic effects on Tregs (Figure 3C,D).

The results of qPCR and flow cytometry showed that the effects of **BPA** and **1** on Tregs were not concentration-dependent, which may be due to the dual regulatory role that flavonoid compounds exhibit in T-cell immunity. According to the reviewed literature, flavonoids exhibit an immune-enhancing effect at low doses, whereas at high doses, they may display an immunosuppressive effect [27]. Given that the components of **BPA** are primarily flavonoids, with compound **1** being the predominant one, we propose that this characteristic may explain why compound **1** and **BPA** did not produce concentration-dependent suppression of regulatory T cell differentiation. In addition, the presence of non-specific components in the extract may also contribute to this phenomenon. There may be trace amounts of undiscovered components in **BPA** that inhibit T-cell immune function, and their effects could become apparent as the concentration of the extract increases.

Tregs are a subset of CD4^+^ T cells characterized by their ineffective immune responses and immunosuppressive capabilities, which enable them to inhibit immune cell-mediated responses [28]. M2 macrophage-secreted cytokines actively suppress CD4^+^ T cell-mediated immune responses [29]. Additionally, IL-10 and TGF-β secreted by Tregs promote the differentiation of M0 macrophages into the M2 subtype, facilitating angiogenesis and establishing an immunosuppressive microenvironment [30]. Furthermore, chemokines secreted by TAMs, such as CCL22, recruit Tregs into the TME, further amplifying immunosuppression [31]. To assess the dynamics of CD4^+^ T cell proliferation, CFDA-SE-labeled CD4^+^ T cells were co-cultured with M2 macrophages or Tregs pretreated with **BPA**, **1** and **3**. A concentration-dependent reversal of M2 macrophage-mediated suppression of CD4^+^ T cells was observed (Figure 4A,B). Additionally, compound **3** reversed CD4^+^ T cell proliferation that had been suppressed by Tregs (Figure 4C,D). In contrast, **BPA** and compound **1** did not significantly affect CD4^+^ T cells proliferation, possibly because they did not alleviate the IL-2 competition between Tregs and CD4^+^ T cells, a known mechanism by which Tregs suppress CD4^+^ T cell proliferation [32]. The above results indicate that **BPA** and its chemical constituents (**1**–**8**) exhibit immunomodulatory effects by suppressing tumor-promoting cells, M2 macrophages and Tregs, thereby potentially exerting antitumor activity by enhanced immune responses.

To validate these findings in a physiologically relevant context, we established a murine colorectal cancer syngeneic model to demonstrate the in vivo anti-tumor efficacy of **BPA**. Consistent with the in vitro studies, **BPA** treatment induced significant reductions in both tumor volume and weight compared to the model control (Figure 5A–C). Furthermore, **BPA** suppressed tumor cell proliferation, as shown by decreased Ki67 expression in IHC assays (Figure 5E,F). Importantly, **BPA** exhibited no apparent toxicity in mice, as reflected by unchanged body weights and normal histological findings in major organs (Figure S5). Interestingly, **BPA** exhibited stronger anti-tumor activity at 50 mg/kg than at 100 mg/kg. As an ethyl acetate extract of *B. pilosa* L., **BPA** is chemically complex; the higher dose may increase hepatic metabolic burden and allow accumulation of low-level toxic constituents that attenuates its intended effect. This aligns with reports that lower doses of plant-derived extracts can improve efficacy, possibly by allowing key bioactive components to act more effectively [33]. For **BPA**, optimized interactions of its constituents at lower concentrations may enhance target engagement while minimizing off-target effects. This underscores the importance of selecting an appropriate dosage for herbal extract treatments to achieve effective therapeutic outcomes, and highlights the need for comprehensive pharmacokinetic and toxicological studies to define safe and efficacious dosing regimens.

The anti-tumor mechanism of **BPA** was further explored in mice. Compared to the model control, **BPA** reduced the levels of IL-10 in mouse serum (Figure 5D), an immunosuppressive cytokine produced by both M2-TAMs and Tregs [34]. This suggests that **BPA** disrupts the cooperation between TAMs and Tregs that inhibits immunosuppression, as documented in the literature [35].

In addition, we also observed that the combination of **BPA** and 5-FU significantly reduce colorectal tumor growth and the effect is better than that of 5-FU alone. 5-FU is a cytotoxic chemotherapeutic agent used clinically for colorectal cancer; however, its effects on cancer are often accompanied by immunosuppression due to bone marrow toxicity. Although significant success has been achieved with tumor immunotherapy, only a minority of patients experience benefits, primarily because of the body’s limited immune response and the intricate, diverse immunosuppressive mechanisms at play [36]. Combining cytotoxic chemotherapy with immunotherapy can effectively decrease tumor burden and suppress production of immunosuppressive factors, thereby enhancing the efficacy of each treatment [37]. Based on this, the combination treatment of **BPA** and 5-FU in mice was established for comparison with 5-FU alone. 5-FU induces immunogenic cell death (ICD), leading to the release of tumor-associated antigens and danger-associated molecular patterns (DAMPs) that promote the recruitment of immune cells, including immunosuppressive subsets, into the TME [38]. In contrast, **BPA** targets the immune microenvironment by suppressing TAMs and Tregs and by promoting the expression of anti-tumor cytokines (Figure 6). This complementary mechanism suggests that **BPA** could counteract the immunosuppressive effects of 5-FU while enhancing its anti-tumor efficacy by restoring immune surveillance within the TME. These findings indicate that **BPA** may serve as an effective immunomodulatory agent, either alone or in combination with conventional chemotherapeutic such as 5-FU, to improve cancer treatment outcomes; however, additional validation is required.

CRC is a prevalent gastrointestinal malignancy characterized by complex pathogenesis influenced by a variety of factors, including environmental changes, genetic variations, and immunity [39]. Among these factors, immune imbalance and inflammatory responses play critical roles in the initiation and progression of CRC [40]. Within the TME, IFN-γ secreted by Th1 cells, CD8^+^ T cells, and NK cells effectively kills tumor cells [41]. However, Tregs and M2-TAMs promote tumorigenesis in CRC by inhibiting Th1 immune responses. Additionally, they limit T-cell trafficking to intestinal tumors via downregulation of endothelial CXCL10 and promote tumor progression through IL-6 expression. Through these and other immunosuppressive mechanisms, Tregs and TAMs, together with their secreted cytokines and chemokines, decrease anti-tumor immunity and are associated with reduced survival in CRC patients [42,43]. To investigate how **BPA** disrupts this network, we examined the mRNA levels of immunosuppressive factors in tumor tissues (Figure 6A). Compared to the model control, **BPA** treatment significantly upregulated the anti-tumor mediators including TNF-α, IL-1β, iNOS, CXCL10 and CCR7. When combined with 5-FU, **BPA** further enhanced the expression of these cytokines, with the exception of IL-1β, which correlated with a greater suppression of tumor growth. Conversely, **BPA** downregulated mRNA expression levels of the pro-tumor cytokines and chemokines such as Foxp3, CD25, CXCR3, CCR4, CCR8, CCR10, TGF-β, Arg-1, YM-1, CCL2, CCL22. Notably, certain immunosuppressive targets, such as CCL22, Foxp3, CCR4, CXCR3, CCR8, were significantly suppressed when combined **BPA** with 5-FU. Moreover, results from immunoblots further confirmed that **BPA** led to a reduction in the expression of immunosuppressive markers (Foxp3, CD25, PD-1, PD-L1, CD206) while increasing the levels of immune stimulatory proteins (CD80, CD4, GITR) in tumor tissues (Figure 6B,C). This divergence likely arises from mechanistic complementarity between **BPA** and 5-FU. **BPA** disrupts crosstalk between Tregs and TAMs by inhibiting IL-10 and CCL22 signaling, while 5-FU induces ICD, releasing DAMPs and antigens to destabilize immunosuppression. Consequently, the remodeling of the TME by 5-FU creates conditions that enhances **BPA**’s ability to suppress immunosuppressive pathways and enhance antitumor immune responses. Herein, experimental evidence (both animal and cellular studies) demonstrate that **BPA** modulates the activity of TAMs and Tregs within the TME, with its low-dose application showing greater therapeutic potential. Further research is warranted to explore these dose-dependent effects and optimize **BPA** formulations to balance efficacy and safety.

This article investigated the tumor immunomodulatory effects of **BPA** and its compounds from both in vivo and in vitro perspectives, and verified the research findings through transcriptome analysis. **BPA** can inhibit the formation of an immunosuppressive microenvironment by suppressing pathways such as IL-17 and NF-κB signaling pathways, thereby reducing the immunosuppressive effects of immune cells (Figure 7). In preliminary studies, we explored the immunomodulatory effects of flavonoids and polyacetylenes from *B. pilosa* L. through network pharmacology and found that they may regulate immune and inflammatory responses through multiple pathways, including the STAT3, AMPK, and NF-κB signaling [44], which is consistent with the findings of this study. Clinically, tumor immunotherapy agents are often used in conjunction with cytotoxic anticancer drugs to achieve synergistic effects through different mechanisms of action. However, the potential for synergistic enhancement between tumor immunotherapy agents that operate through different mechanisms is worthy of further exploration. As the key immunotherapeutic agents for cancer, inhibitors of the immune checkpoint PD-1/PD-L1 primarily restore T-cell immune function by blocking the PD-1/PD-L1 signaling pathway, thereby facilitating tumor cell destruction [45]. In our study, we observed that **BPA** treatment reduced the expression of PD-1 and PD-L1 in tumor tissues. These results prompt the hypothesis that **BPA** could be used in combination with PD-1 or PD-L1 inhibitors to augment their antitumor activity. Further preclinical investigations, particularly comprehensive pharmacokinetic analyses, rigorous toxicology assessments, and tumor re-challenge studies, are required to fully evaluate the efficacy and safety of these combination regimens.

## 5. Conclusions

In summary, **BPA**, an extract derived from *B. pilosa* L. and enriched in flavonoids, shows anti-tumor effects in mice by suppressing macrophages polarization toward M2-like TAMs and inhibiting the differentiation of Naïve CD4^+^ T cells into immunosuppressive Tregs within the TME. This promotes the activation and proliferation of effector CD4^+^ T cells. These findings provide a theoretical basis for the clinical anticancer use of the traditional medicinal herb *B. pilosa* L. and suggest that **BPA** suppresses pro-tumor immune cells, indicating antitumor efficacy in the treatment of CRC. Although **BPA** demonstrated immunotherapeutic effects, comprehensive preclinical studies to define optimal dosing, pharmacodynamics, and mechanisms of action are needed to validate its potential in tumor immunotherapy.

## Data Availability

The original contributions presented in this study are included in the article/Supplementary Materials. Further inquiries can be directed to the corresponding author.

## Supplementary Materials

The following supporting information can be downloaded at: https://www.mdpi.com/article/10.3390/cells15020126/s1, Table S1: Chemical information of compounds **1**–**8**; Table S2: Primer sequences used for qRT-PCR analysis; Table S3: Antibody information utilized in the Western blot experiment; Figure S1: The effects of **BPA** and **1**–**8** on M1/M2 macrophages and Tregs analyzed by qRT-PCR. (**A**) Arg-1 mRNA expression in M2 macrophages; (**B**) CD25 mRNA expression in Tregs; (**C**) iNOS mRNA expression in M1 macrophages; (**D**) IL-1β mRNA expression in M1 macrophages. Gene expression levels were quantified using qRT-PCR and normalized to 18SRNA as the reference gene. Statistical significance for all experiments is denoted as follows (n = 3): compared to the M0 or NaÏve group, ^#^ *p* < 0.05, ^##^ *p* < 0.01 and ^####^ *p* < 0.0001; compared to M2/M1/Tregs group, * *p* < 0.05, ** *p* < 0.01, *** *p* < 0.001, and **** *p* < 0.0001; Figure S2: Flow cytometry plots for macrophage gating strategy and CD4^+^ T cell purity. (**A**) Gating strategy used for flow cytometric identification of macrophages. (**B**) Purity of CD4^+^ T cells. Left, CD4^+^ T cell purity among lymphocytes isolated from mouse lymph node and spleen. Right, CD4^+^ T cell purity after isolation using a CD4^+^ T cell isolation kit; Figure S3: Effects of **BPA** and **1**–**8** on M0 differentiation into M2 macrophages and CD4^+^ T cell differentiation into Tregs. (**A**) Representative flow cytometry plots of CD25^+^Foxp3^+^. (**B**) Quantification of CD25^+^Foxp3^+^ frequency. (**C**) Representative flow cytometry plots of F4/80^+^CD206^+^. (**D**) Quantification of F4/80^+^CD206^+^ frequency. (**E**) Representative flow cytometry plots of F4/80^+^CD80^+^. (**F**) Quantification of F4/80^+^CD80^+^ frequency. (**G**) Representative flow cytometry plots of CD206^+^/CD80^+^. (**H**) Quantification of CD206^+^/CD80^+^ frequency. Statistical significance for all experiments is denoted as follows (n = 3): compared to the M0/Naïve group, ^####^ *p* < 0.0001; compared to M2 /Tregs group, * *p* < 0.05, ** *p* < 0.01, *** *p* < 0.001, and **** *p* < 0.0001. RFI means Relative Fluorescence Intensity; Figure S4: Dose-dependent effects of **BPA**, **1**, and **3** on M2 macrophages differentiation. (**A**) Flow cytometry analysis of F4/80^+^CD80^+^ under increasing concentrations of **BPA** (12.5, 25, 50 μg/mL) or 1 and 3 (12.5, 25, 50 μM). (**B**) The histograms for the quantified results of F4/80^+^CD80^+^ cells. (**C**) Analysis of F4/80^+^CD206^+^ cell population using flow cytometry. (**D**) The histograms for the quantified results of F4/80^+^CD206^+^ cells. Statistical significance for all experiments is denoted as follows (n = 3): compared to the M0 group, ^#^ *p* < 0.05, ^###^ *p* < 0.001, and ^####^ *p* < 0.0001; compared to M2 group, * *p* < 0.05, ** *p* < 0.01, and **** *p* < 0.0001. RFI means Relative Fluorescence Intensity; Figure S5: Body weight changes and representative H&E-stained images of mouse organs. (**A**) Statistical analysis of mouse body weight across all treatment groups. (**B**) Histopathological analysis of mouse visceral tissues (heart, liver, spleen, lungs, kidneys, and stomach) using H&E staining, indicating the absence of significant toxicity in the treatment groups. Magnified 200 times.

## Abbreviations

The following abbreviations are used in this manuscript:

TME	The tumor microenvironment
TAMs	Tumor-associated macrophages
Tregs	Regulatory T cells
MDSCs	Myeloid-derived suppressor cells
DCs	Dendritic cells
NK	Natural killer cells
IFN-γ	Interferon-γ
Arg-1	Arginase-1
iNOS	Inducible nitric oxide synthase
qRT-PCR	Quantitative real-time PCR
5-FU	5-Fluorouracil
IL-1β	Interleukin-1β
IHC	immunohistochemistry
CRC	Colorectal cancer
FCM	Flow cytometry
