# Supplementary material for: Cancer Immunomodulatory Effect of Bidens pilosa L. in Mice: Suppression of Tumor-Associated Macrophages and Regulatory T Cells
Source: Cells. 2026 Jan 10;15(2):126. doi: 10.3390/cells15020126 (PMC12838707; doi:10.3390/cells15020126)
Supplement: Supplementary file 1 [file cells-15-00126-s001.zip › cells-4061615-supplementary.pdf]

Supporting Information

Cancer Immunomodulatory Effect of *Bidens pilosa* L. in Mice: Suppression of Tumor-Associated Macrophages and Regulatory T cells

Meihua Zhu[1,2,†], Jiayan Xiong[1,2,†], Ruyi Zhang[1], Xingyan Yang[1], Weiqing Sun[1], Ziyi Yang[1,2], Yuhan Chai[1,2], Yang Tao[1,2], Yu-Qiang Zhao[1], Baomin Fan[1,2],and Guangzhi Zeng[1,2,*]

[1] Yunnan Key Laboratory of Chiral Functional Substance Research and Application, Yunnan Minzu University, Kunming 650504, China

[2] Key Laboratory of Chemistry in Ethnic Medicinal Resources, State Ethnic Affairs Commission and Ministry of Education, Yunnan Minzu University, Kunming 650504, China;

*Correspondence: g.zh_zeng@ymu.edu.cn(G.-Z. Zeng)

[†] These authors contributed equally to this work.

*Correspondence e-mail addresses: No. 2929, Yuehua Street, Chenggong District, Kunming City, Yunnan Province, China. Tel: 86-871-65946920, Fax: 86-871-65946330;

Contents

Table S1. Chemical information of compounds **1**-**8** 3

Table S2. Primer sequences used for qRT-PCR analysis 4

Table S3. Antibody information utilized in Western blot experiment 5

Figure S1. Effects of **BPA** and **1**-**8** on M1/M2 macrophages and Tregs 6

Figure S2. Flow cytometry plots for macrophage gating strategy and CD4+ T cell purity 7

Figure S3. Effects of **BPA** and **1**-**8** on M0 differentiation into M2 macrophages and $CD4^+$ T cell differentiation into Tregs 8

Figure S4. Dose-dependent effects of **BPA**, **1**, and **3** on M2 macrophages differentiation 9

Figure S5. Body weight changes and representative H&E-stained images of mouse organs 10

Table S1. Chemical information of compounds **1**-**8**.

Compound	Chemical Name
**1**	8,3'-dihydroxy-3,7,4'-trimethoxy-6-O-β-D-glucopyranosyl flavone
**2**	3-β-D-Glucopyranosyloxy-1-hydroxy-6(E)-tetradecene-8,10,12-triyne
**3**	3, 5-dihydroxy-3´, 5´-dimethoxyflavon-7-O-β-D-glucopyranoside
**4**	Trifolin
**5**	3, 6-O-dimethylquercetagetin-7-O-β-D-glucoside
**6**	3,4'-dimethoxy-7-O-β-glucopyranoside quercetin
**7**	hesperetin-7-O-β-D-glucopyranoside
**8**	luteolin

Table S2. Primer sequences used for qRT-PCR analysis.

Mouse Genes	Forward Primer (5'-3')	Reverse Primer (5'-3')
**ARG1**	CTCCAAGCCAAAGTCCTTAGAG	GGAGCTGTCATTAGGGACATCA
**CD25**	GAAGGCATACCAGAAAGGGTTGA	ACTCTGTCCTTCCACGAAATGAT
**YM1**	CATGAGCAAGACTTGCGTGAC	GGTCCAAACTTCCATCCTCCA
**INOS**	GTTCTCAGCCCAACAATACAAGA	GTGGACGGGTCGATGTCAC
**TNFA**	CAGGCGGTGCCTATGTCTC	CGATCACCCCGAAGTTCAGTAG
**CCL2**	ATGCAGGTCCCTGTCATGCTTCTG	CTAGTTCACTGTCACACTGGTCACTCC
**CCL22**	TCTTGCTGTGGCAATTCAGA	GAGGGTGACGGATGTAGTCC
**IL-10**	ATCGATTTCTCCCCTGTGAA	TGTCAAATTCATTCATGGCCT
**IL-1B**	GAAATGCCACCTTTTGACAGTG	TGGATGCTCTCATCAGGACAG
**CXCL10**	CCAAGTGCTGCCGTCATTTTC	GGCTCGCAGGGATGATTTCAA
**CCR7**	CATTGCCTATGACGTCACCTACA	GAAGGCATACCAGAAAGGGTTGA
**FOXP3**	TTCCTCCCGCTCTCTGACTCT	AAGCGCCAGTTGTGTACAAATATC
**TGFB**	CCACCTGCAAGACCATCGAC	CTGGCGAGCCTTAGTTTGGAC
**CXCR3**	AATGCCACCCATTGCCAGTAC	AGCAGTAGGCCATGACCAGAAG
**CCR4**	TGCACCAAGGAAGGTATCAAGG	GTACACGTCCGTCATGGACTT
**CCR8**	CGTGGGCTGCAAGAAACTGA	AGAGACCACCTTACACATCGC
**CCR10**	CTGACCTTTTATTGGCCCTGAC	CGAAGACTGAAACCAAGTGCG
**VEGFR2**	CTGGAGCCTACAAGTGCTCG	GAGGTTTGAAATCGACCCTCG
**18S rRNA**	CAGCCACCCGAGATTGAGCA	TAGTAGCGACGGGCGGGTGT

Table S3. Antibody information utilized in the Western blot experiment

Antibody	Dilution	Company
CD206	1:6000	Proteintech, cat. #81525-1-RR
CD80	1:4000	Proteintech, cat. #66406-1-Ig
Foxp3	1:6000	Proteintech, cat. #22228-1-AP
CD25	1:5000	ABclonal, cat. #A2984
PD-1	1:10000	Proteintech, cat. #66220-1-Ig
PD-L1	1:15000	CUSABIO, cat. #CSB-MA878942A1m
GITR	1:5000	Proteintech, cat. #23899-1-AP
CD4	1:3000	Proteintech, cat. #19068-1-AP
VEGFR2	1:2000	Proteintech, cat. #26415-1-AP
GAPDH	1:10000	Proteintech, cat. #60004-1-Ig
HRP Aipha Tubulin Mouse Monoclonal Antibody	1:10000	Proteintech, cat. #HRP-66031
HRP-conjugated Affinipure Goat anti-Rabbit IgG(H+L)	1:10000	Proteintech, cat. #RGAR001
HRP-conjugated Affinipure Goat anti-Mouse IgG(H+L)	1:10000	Proteintech, cat. #RCAM001

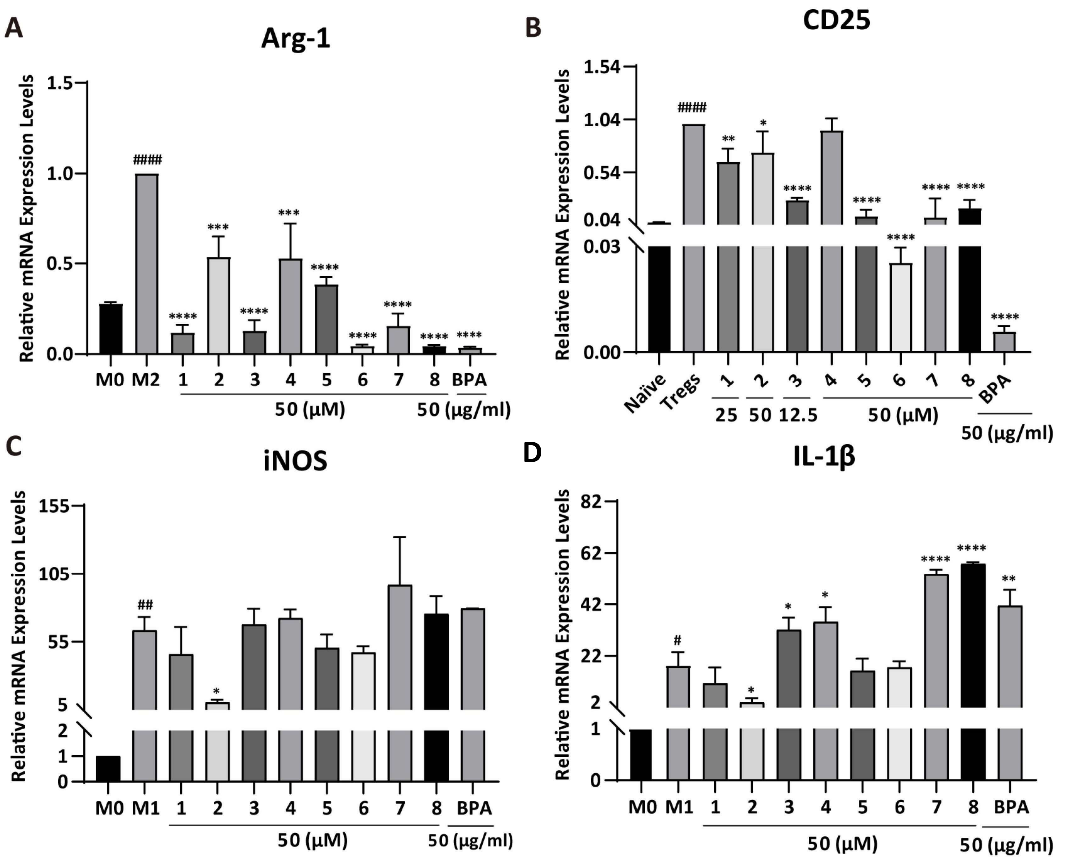

Figure S1. The effects of **BPA** and **1-8** on M1/M2 macrophages and Tregs analyzed by qRT-PCR. (**A**) Arg-1 mRNA expression in M2 macrophages; (**B**) CD25 mRNA expression in Tregs; (**C**) iNOS mRNA expression in M1 macrophages; **(D)** IL-1β mRNA expression in M1 macrophages. Gene expression levels were quantified using qRT-PCR and normalized to 18SRNA as the reference gene. Statistical significance for all experiments is denoted as follows(n=3): compared to the M0 or Naïve group, $^{\#}$*P*<0.05, $^{\#\#}$*P*<0.01 and $^{\#\#\#\#}$*P*<0.0001; compared to M2/M1/Tregs group, **P*<0.05, ** *P*<0.01, ****P*<0.001, and **** *P*<0.0001.

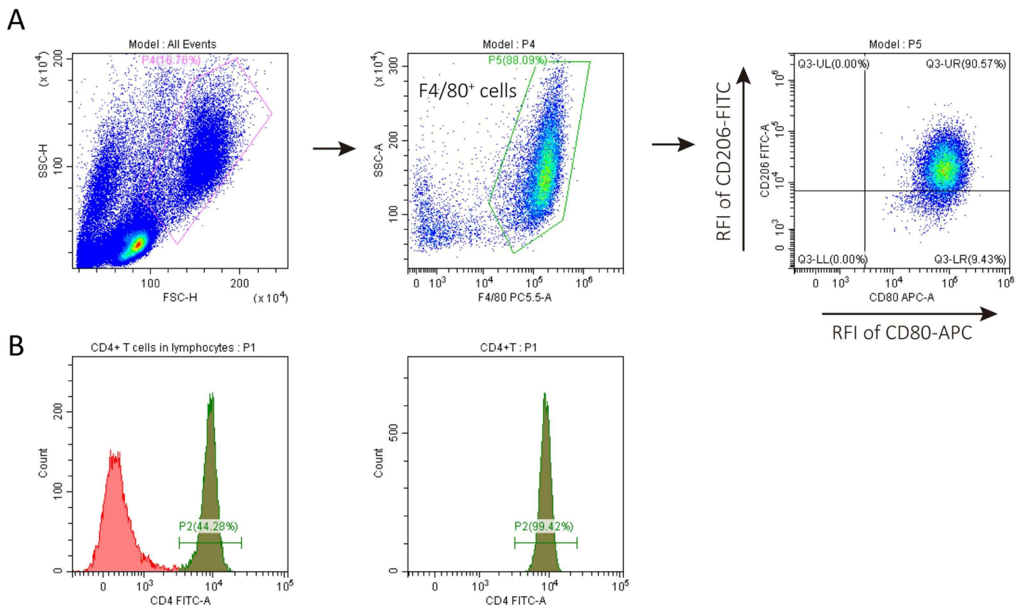

Figure S2. Flow cytometry plots for macrophage gating strategy and CD4⁺ T cell purity. (A) Gating strategy used for flow cytometric identification of macrophages. (B) Purity of CD4⁺ T cells. Left, CD4⁺ T cell purity among lymphocytes isolated from mouse lymph node and spleen. Right, CD4⁺ T cell purity after isolation using a CD4⁺ T cell isolation kit.

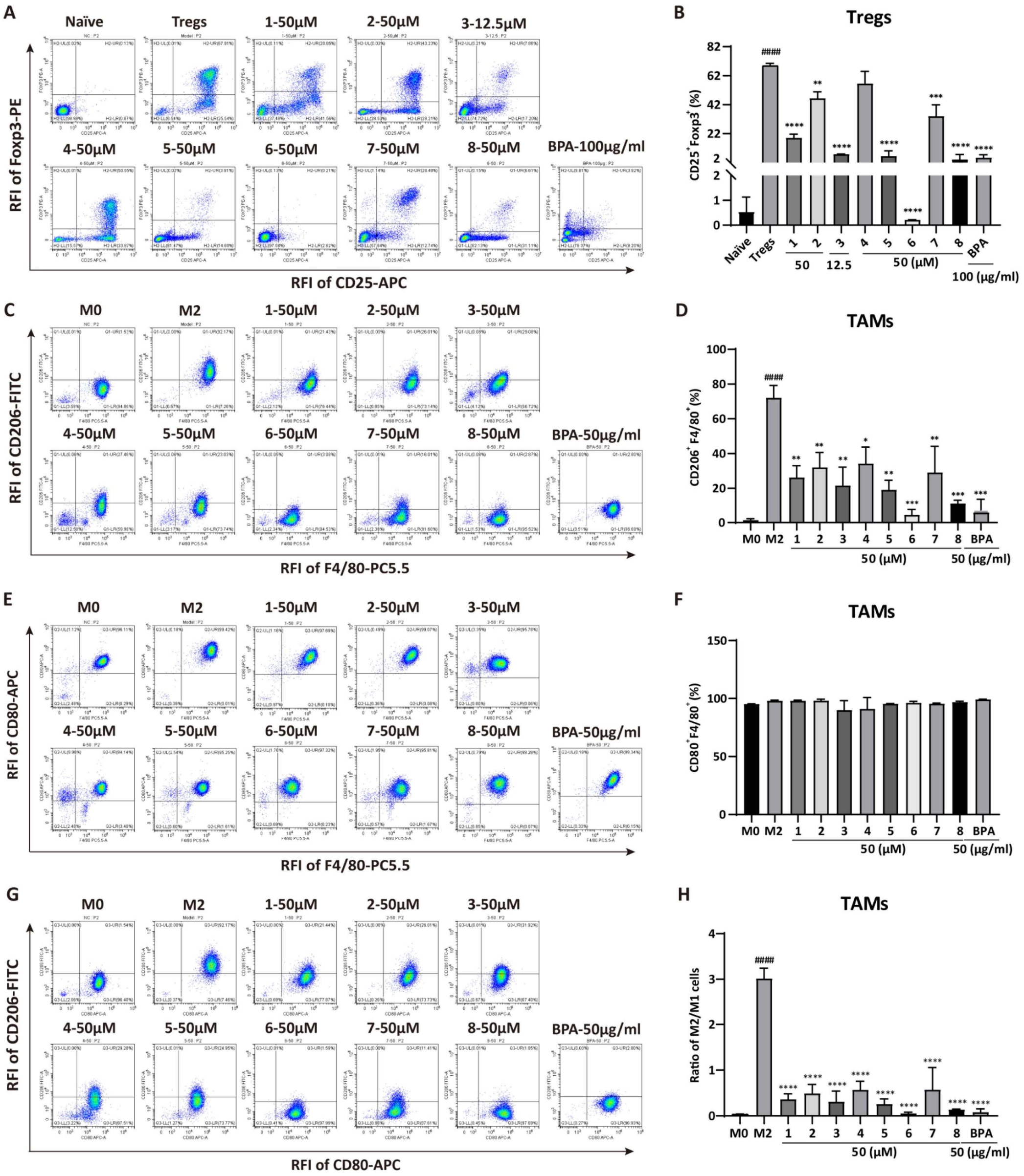

Figure S3. Effects of **BPA** and **1**-**8** on M0 differentiation into M2 macrophages and CD4+ T cell differentiation into Tregs. (**A**) Representative flow cytometry plots of CD25+Foxp3+. (**B**) Quantification of CD25+Foxp3+ frequency. (**C**) Representative flow cytometry plots of F4/80+CD206+. (**D**) Quantification of F4/80+CD206+ frequency. (**E**) Representative flow cytometry plots of F4/80+CD80+. (**F**) Quantification of F4/80+CD80+ frequency. (**G**) Representative flow cytometry plots of CD206+/CD80+. (**H**) Quantification of CD206+/CD80+ frequency. Statistical significance for all experiments is denoted as follows (n=3): compared to the M0/Naïve group, ####*p*<0.0001; compared to M2 /Tregs group, **p*<0.05, ** *p*<0.01, *** *p*<0.001, and **** *p*<0.0001. RFI means Relative Fluorescence Intensity.

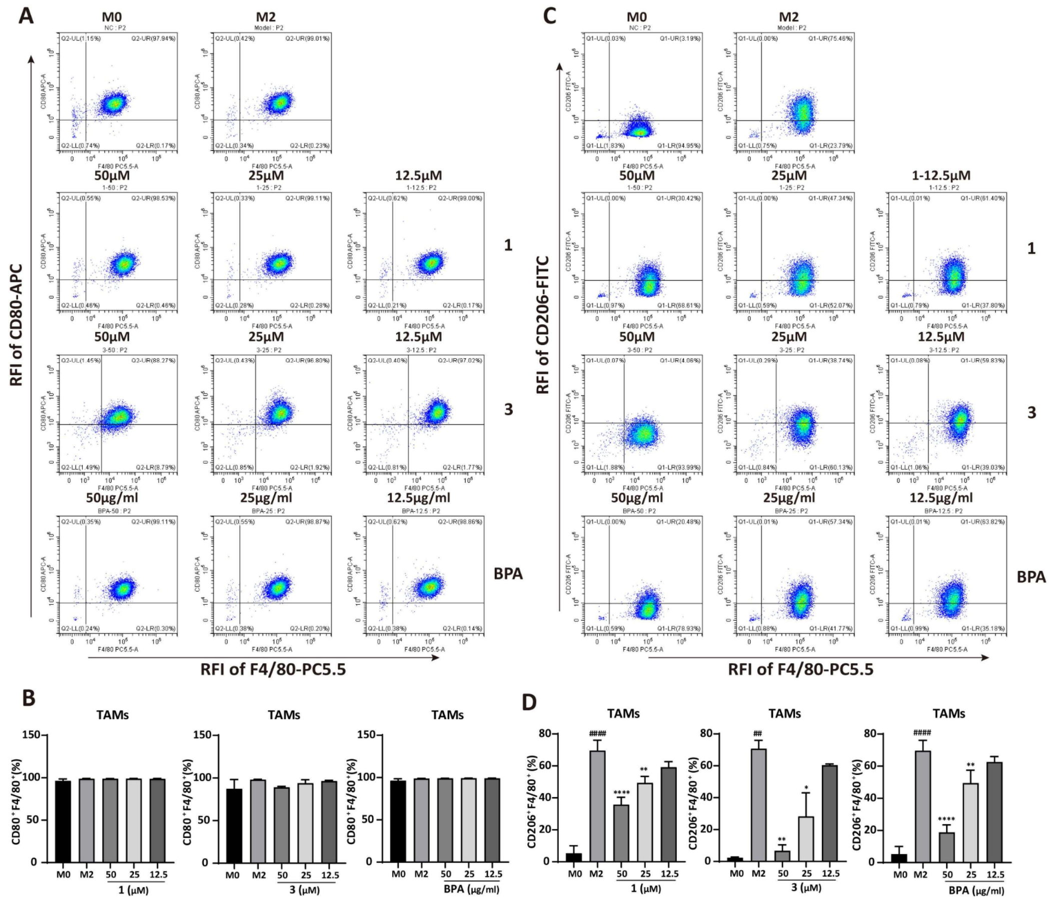

Figure S4. Dose-dependent effects of **BPA**, **1**, and **3** on M2 macrophages differentiation. (**A**) Flow cytometry analysis of F4/80$^+$CD80$^+$ under increasing concentrations of BPA (12.5, 25, 50 μg/mL) or **1** and **3** (12.5, 25, 50 μM). (**B**) The histograms for the quantified results of F4/80$^+$CD80$^+$ cells. (**C**) Analysis of F4/80$^+$CD206$^+$ cell population using flow cytometry. (**D**) The histograms for the quantified results of F4/80$^+$CD206$^+$ cells. Statistical significance for all experiments is denoted as follows (n=3): compared to the M0 group, $^{\#}p<0.05$, $^{\#\#\#}p<0.001$, and $^{\#\#\#\#}p<0.0001$; compared to M2 group, $^{*}p<0.05$, $^{**}p<0.01$, and $^{****}p<0.0001$. RFI means Relative Fluorescence Intensity.

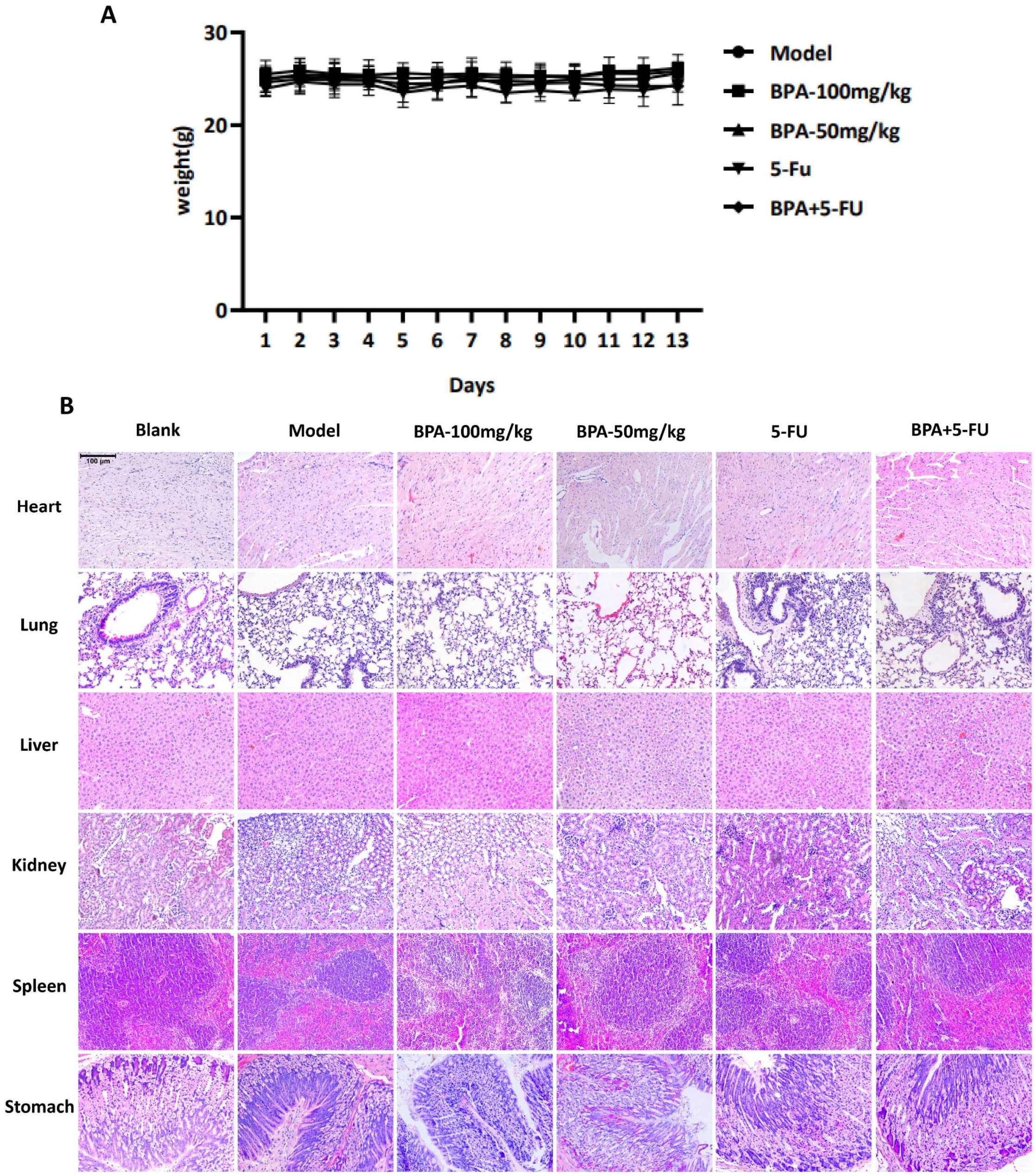

Figure S5. Body weight changes and representative H&E-stained images of mouse organs. (**A**) Statistical analysis of mouse body weight across all treatment groups. (**B**) Histopathological analysis of mouse visceral tissues (heart, liver, spleen, lungs, kidneys, and stomach) using H&E staining, indicating the absence of significant toxicity in the treatment groups. Magnified 200 times.
